# Neither too much, neither too little! Positive fluid balance and ICU outcome

**DOI:** 10.1186/cc14710

**Published:** 2015-09-28

**Authors:** Allison EPP Borges, Carlos Augusto R Feijó, Eduardo Q da Cunha, Francisco A de Meneses, Marina P Albuquerque, Natália LP Aragão, Tamara O Pinheiro, Túlio S de Aguiar

**Affiliations:** 1 General Hospital of Fortaleza, Papicu, Fortaleza, SP, Brazil

## Introduction

The fluid balance of critically ill patients has emerged as a potential marker of disease severity. This is associated with worse outcome and prolonged time of use of intensive care support in the ICU.

## Objective

To research the influence of positive fluid balance in the first 72 hours of hospitalization in the ICU on organ dysfunction and outcome.

## Methods

Retrospective study including patients admitted to the General Hospital of Fortaleza/SESA ICU, from November 2014 to February 2015. Patients were characterized by the presence of circulatory and renal dysfunction at the discretion of the SOFA score. The days of mechanical ventilation were computed. The multivariate analysis was performed by ANOVA test.

## Results

From a total of 86 patients, 51 % were men, the mean age was 53.95 ± 19.99 years, and mean APACHE II score was 14.47 ± 7.2 points. Of these, 68 patients (79 %) had a fluid balance measured in the first 72 hours of admission and were included in the study. The fluid balance was higher in clinical patients, rather than surgical patients (4183.86 vs. 2491.88 ml; *p *= 0.049). Patients who did not use mechanical ventilation had lower values of positive fluid balance compared with those who used that support (1687.47 vs. 4499.5 ml, *p *= 0.02). There was a more meaningful fluid overload in patients with renal and cardiovascular dysfunctions than in those without these disorders ((4317.59 vs. 3465.61 ml; *p *= 0.09); (3184.95 vs. 4405.23 ml; *p *= 0.092), respectively). The length of stay in the ICU for those patients who had fluid balance greater than 2000 ml in the first 72 hours was 16.06 days, while for those with fluid balance <2000 ml was 9.1 days (*p *= 0.041). There were differences between the fluid balance values of patients who died and those who were discharged from the ICU, but without statistical significance.

## Conclusion

Our findings show that fluid overload with positive cumulative fluid balance during the first 72 hours in the ICU is associated with longer ICU length of stay, but without impact on ICU mortality.

